# Protein Phosphatase 6 Protects Prophase I-Arrested Oocytes by Safeguarding Genomic Integrity

**DOI:** 10.1371/journal.pgen.1006513

**Published:** 2016-12-08

**Authors:** Meng-Wen Hu, Tie-Gang Meng, Zong-Zhe Jiang, Ming-Zhe Dong, Heide Schatten, Xingzhi Xu, Zhen-Bo Wang, Qing-Yuan Sun

**Affiliations:** 1 State Key Laboratory of Stem Cell and Reproductive Biology, Institute of Zoology, Chinese Academy of Sciences, Beijing, China; 2 University of Chinese Academy of Sciences, Beijing, China; 3 Department of Veterinary Pathobiology, University of Missouri, Columbia, MO, United States of America; 4 Beijing Key Laboratory of DNA Damage Response and College of Life Sciences, Capital Normal University, Beijing, China; University of Pennsylvania, UNITED STATES

## Abstract

Mammalian oocytes are arrested at prophase of the first meiotic division in the primordial follicle pool for months, even years, after birth depending on species, and only a limited number of oocytes resume meiosis, complete maturation, and ovulate with each reproductive cycle. We recently reported that protein phosphatase 6 (PP6), a member of the PP2A-like subfamily, which accounts for cellular serine/threonine phosphatase activity, functions in completing the second meiosis. Here, we generated mutant mice with a specific deletion of *Ppp6c* in oocytes from the primordial follicle stage by crossing *Ppp6c*^*F/F*^ mice with *Gdf9-Cre* mice and found that *Ppp6c*^*F/F*^*; GCre+* mice are infertile. Depletion of PP6c caused folliculogenesis defects and germ cell loss independent of the traditional AKT/mTOR pathway, but due to persistent phosphorylation of H2AX (a marker of double strand breaks), increased susceptibility to DNA damage and defective DNA repair, which led to massive oocyte elimination and eventually premature ovarian failure (POF). Our findings uncover an important role for PP6 as an indispensable guardian of genomic integrity of the lengthy prophase I oocyte arrest, maintenance of primordial follicle pool, and thus female fertility.

## Author Summary

Formation of haploid gametes from diploid germ cells requires a specialized reductive cell division known as meiosis. In contrast to male meiosis that takes place continuously, a unique feature of female meiosis in mammals is the long arrest in meiosis I, which lasts up to 50 years in humans. Because the size of the germ cell pool determines the reproductive lifespan of females, it is important to discover mechanisms preserving the germ cell pool during the lengthy meiotic arrest. In this study, we examined the physiological role of a member of the PP2A-like serine/threonine phosphatase subfamily, protein phosphatase 6, in mouse oocytes during ovarian follicular development. This is the first study linking PP6 to the maintenance of the female germ cell pool and fertility. We find PP6 is an indispensable protector of arrested oocytes by safeguarding genomic integrity during their dormancy in the mouse ovary.

## Introduction

In mammals, females are born with a finite number of oocytes contained within primordial follicles that serve as the source of ova for the entire period of reproductive life. To produce mature eggs, dormant primordial follicles are recruited into the growing follicle pool, a process termed as initial follicular recruitment or activation. Activated follicles subsequently develop into primary follicles, secondary follicles, and antral follicles [1]. Throughout this follicular growth process, oocytes grow while being arrested in prophase of meiosis I with homologs held together by chiasmata. Only a few dominant antral follicles reach the preovulatory stage and release a mature egg for fertilization after a gonadotropin surge during each estrus cycle [2]. When the ovarian follicle reserve is exhausted in women, menopause occurs. However, disorders during folliculogenesis could lead to follicle depletion in advance and cause premature ovarian insufficiency (POI) or premature ovarian failure (POF), which is a main cause of female infertility in humans and affects nearly 1% women under the age of 40 [3].

Protein phosphorylation, mediated by a conserved cohort of protein kinases and phosphatases, regulate follicular activation and growth, meiotic cell cycle arrest and progression, chromosome dynamics, and ovulation [4]. Numerous studies using genetically modified mice reveal that protein kinases play important roles during folliculogenesis/oogenesis. For example, the PTEN/PI3K/AKT signaling pathway regulates follicular activation and survival [5]. Recently, we reported that LKB1 acts as a gatekeeper of the ovarian primordial follicle pool [6]. In contrast, there is limited information about the roles of protein phosphatases. Among the serine/threonine phosphoprotein phosphatases (PPPs), PP2A, PP4 and PP6 form a subfamily called PP2A-like protein phosphatases, which share high homology in the catalytic subunit and account for the majority of cellular serine/threonine phosphatase activity [7, 8]. PP2A is involved in regulating chromosome condensation, DNA damage repair, G2/M transition and sister chromatid cohesion [9]. Our recent knockout mouse model revealed that oocyte PP2A is dispensable for folliculogenesis, though PP2A has been reported to dephosphorylate AKT and AMPK, important kinases for folliculogenesis [10]. Although PP6 was discovered almost 20 years ago, progress has been slow regarding its functions in cells, not to mention its specific functions in meiotic cells.

The PP6 holoenzyme consists of a catalytic subunit, PP6c, one of the three regulatory subunits including SAPS1, 2, 3 (also known as PP6R1, PP6R2 and PP6R3, respectively), and one of the three ankyrin repeat subunits including ARS-A, -B, -C [11, 12]. PP6 is conserved among all eukaryotic species from yeast to humans, attesting to its fundamental importance. Mutations in PP6c are found to exist in 9–12.4% melanomas surveyed and may act as drivers for melanoma development [13, 14]. The PP6 yeast homologue, Sit4/Ppe1, is required for G1/S progression and equal chromosome segregation [15, 16], and plays a role in signaling through the target of rapamycin (TOR), a key nutrient-sensing kinase [17]. Human PP6 has an established role in DNA damage response with its ability to modulate signaling by DNA-dependent protein kinase (DNA-PK), homology recombination-mediated repair of DNA double strand breaks (DSBs) [18, 19], as well as its interactions with Aurora A kinase [20, 21]. More recent studies suggest a broader role for PP6 in pre-mRNA splicing [22], control of apoptosis in immune cells [23], formation of adherens junctions through interaction with E-cadherin [24], and modulation of signaling through the Hippo pathway [25]. Overall, these data suggest that PP6 integrates signaling from multiple pathways.

Genetically modified mouse models are powerful tools for studying gene function *in vivo* [26, 27]. We recently reported that a conditional knockout of PP6 in oocytes from growing follicles (by crossing *Ppp6c*^*F/F*^ mice with *Zp3-Cre* mice) causes female subfertility by disrupting MII spindle organization and MII completion after fertilization [28]. Here, we crossed *Ppp6c*^*F/F*^ mice with *Gdf9-Cre* mice to generate mutant mice with specific deletion of *Ppp6c* in prophase I-arrested oocytes from the primordial follicle stage. We find that PP6 plays a critical role in germ cell survival and follicular development by safeguarding genomic integrity of prophase I-arrested oocytes.

## Results

### PP6c is essential for female fertility

**Fig 1:**
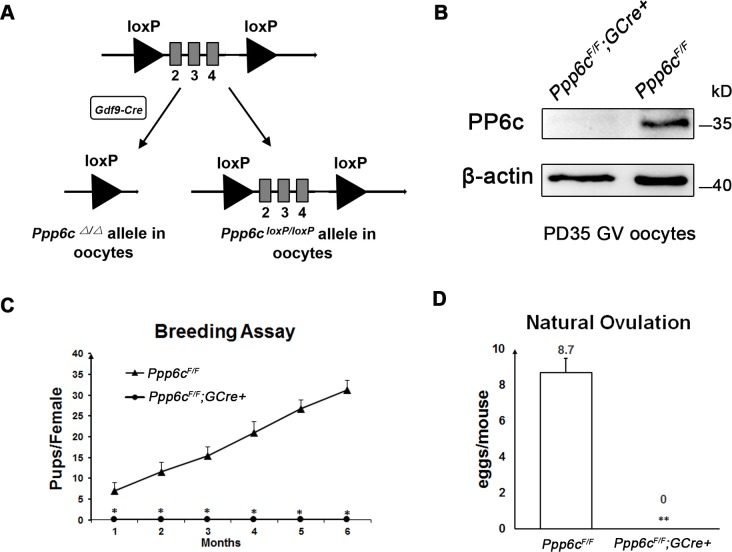
PP6c is essential for female fertility. (A) Schematic representation of deletion of *Ppp6c* exons and creation of *Ppp6c* Δ allele by *Gdf9-Cre*-mediated recombination in oocytes. (B) Western blots showing the absence of PP6c protein expression in *Ppp6c*^*F/F*^*;GCre+* oocytes. The amount of β-actin was used as an internal control. Molecular mass is given in kilodaltons. Oocytes were isolated from ovaries of PD35 mice and used for western blotting. For each lane, 200 GV oocytes were used. For each experiment, at least 5 mice of each genotype were used. (C) Infertility of the female ^*F/F*^*;GCre+* mice. Continuous breeding showed the cumulative number of progeny per female mouse for 6 months. At least 6 mice of each genotype were used. (D) Anovulation of *Ppp6c*^*F/F*^*; GCre+* female mice. Fertilized eggs were collected and counted from females with vaginal plugs after mating. At least 6 mice of each genotype were used.

To explore the *in vivo* roles of PP6 during folliculogenesis/oogenesis, we generated mutant mice (referred to as *Ppp6c*^*F/F*^*;GCre+* mice), in which exon II-IV of the *Ppp6c* gene were targeted, by crossing *Ppp6c*^*F/F*^ mice [28] with transgenic mice expressing *Gdf9* promoter-mediated Cre recombinase [5] (Fig 1A). In *Gdf9-Cre* mice, Cre is specifically expressed in oocytes of primordial follicles and later stage follicles since postnatal day 3 [27]. By immunoblotting analysis, we confirmed successful depletion of PP6c protein in GV oocytes from *Ppp6c*^*F/F*^*;GCre+* females (Fig 1B).

To investigate the effect of oocyte-specific knockout of PP6c on female fertility, a breeding assay was carried out by mating *Ppp6c*^*F/F*^ or *Ppp6c*^*F/F*^*;GCre+* female mice with males of proven fertility for 6 months. As shown in Fig 1C, female *Ppp6c*^*F/F*^*;GCre+* mice were completely infertile. The infertility appeared to be due to anovulation in adult mutant mice, whereas control mice ovulated normal numbers of eggs (8.7±0.8) in the natural ovulation assays (Fig 1D).

### Depletion of PP6c in oocytes during the primordial follicle stage leads to premature ovarian failure because of primordial follicle arrest and growing follicle atresia

**Fig 2:**
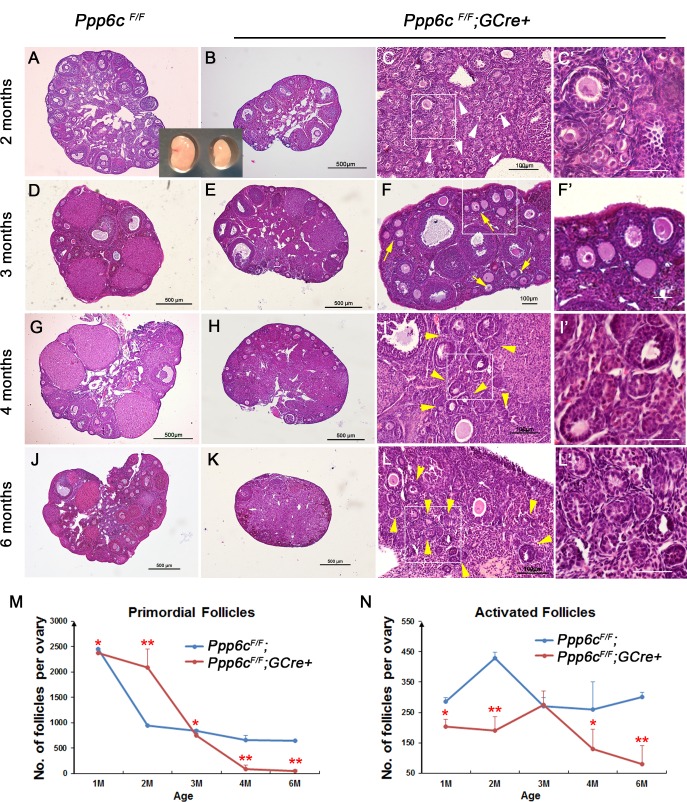
Premature ovarian failure in *Ppp6c*^*F/F*^*;GCre+* mice. (A-L) Histology of ovarian sections from *Ppp6c*^*F/F*^ and *Ppp6c*^*F/F*^*;GCre+* females of 2 months, 3 months, 4 months and 6 months-of-age, respectively, stained with hematoxylin and eosin. White arrowheads in C point to primordial follicles; yellow arrows in F show activated follicles; yellow arrowheads in I and L indicate atretic follicles. Panels C’, F’, I’ and L’ are magnified images of rectangular areas marked with a solid line in panels C, F, I and L, respectively. Bars: 100 μm in C, F, I and L; 50 μm in C’, F’, I’ and L’; 500 μm in the others. For each time point, at least 3 mice of each genotype were used for analysis, and representative images are shown. (M-N) Numbers of primordial follicles (M) and activated follicles (N) in ovaries of 1-month (1 mo), 2-month (2 mo), 3-month (3 mo), 4-month (4 mo) and 6-month (6 mo)-old *Ppp6c*^*F/F*^ and *Ppp6c*^*F/F*^*;GCre+* females. For each time point, at least 3 mice of each genotype were used for analysis. Data are shown as mean ± SEM.*P< 0.05; **P< 0.01.

To understand the defects of the mutant mice, we first observed the morphology of ovaries from both *Ppp6c*^*F/F*^ and *Ppp6c*^*F/F*^*;GCre+* mice. At 1 month-of-age, both histological morphology of ovaries and numbers of follicles were similar between *Ppp6c*^*F/F*^ and *Ppp6c*^*F/F*^*;GCre+* ovaries (S1A and S1B Fig), indicating that comparable numbers of follicles are formed in the wild-type and mutant ovaries. However, after 1 month-of-age, the time of onset of sexual maturity, mutant ovaries started to show differences and became smaller than the controls. In ovaries of 2-month-old *Ppp6c*^*F/F*^*;GCre+* mice, there were few growing follicles (Fig 2B) in contrast to control ovaries that contained many healthy-looking growing follicles (Fig 2A). However, large clusters of primordial follicles could still be observed on the ovarian surface area of 2-month-old mutant ovaries (white arrowheads, Fig 2C and 2C’), compared with control ovaries where such clusters barely could be found. Consistently, the number of primordial follicles in 2-month-old *Ppp6c*^*F/F*^*;GCre+* ovaries was more than double that of *Ppp6c*^*F/F*^ ovaries (Fig 2M and S1C Fig). The numbers of large growing follicles, especially Type 5 and Type 6 follicles, were significantly decreased, corresponding to only 16.7% and 36.5% of those in control ovaries (S1C Fig). At 3 months-of-age, clusters of primordial follicles were no longer observed on the ovarian surface; instead, many primary follicles appeared in the same location, indicating delayed activation of the arrested primordial follicles (yellow arrows, Fig 2F and 2F’). Consistent with these observations, quantification of ovarian follicles revealed a significant reduction of primordial follicles and an increase in type 3 and type 4 follicles in 3-month-old mutant ovaries (S1D Fig). Nevertheless, large growing follicles (including type 5 and type 6 follicles) were significantly fewer than those in control ovaries (S1D Fig), though both control and mutant ovaries might contain similar numbers of primordial follicles and activated follicles (Fig 2M and 2N). These later activated follicles, however, could not serve as the source of ova for mutant mice, probably because they died soon after activation with only empty follicle-like structures left at the ovarian surface (yellow arrowheads, Fig 2I, 2I’, 2L and 2L’). At 4 months-of-age, only a few primary follicles and small secondary follicles were seen at the cortical region of mutant ovaries (Fig 2H), and the other types of follicles (including primordial follicles, type 5,6 and 7 follicles) were disappearing (S1E Fig). By 6 months-of-age, almost all types of follicles were depleted in *Ppp6c*^*F/F*^*;GCre+* ovaries (Fig 2K and 2L; S1F Fig), which is termed POF. In general, from 1 month to 2 months postpartum, more than half of the primordial follicles in the *Ppp6c*^*F/F*^ ovaries decreased due to both follicular activation and atresia. In contrast, loss of primordial follicles in *Ppp6c*^*F/F*^*;GCre+* ovaries was slower because they failed to be activated upon puberty and stayed arrested until 2 months postpartum, after which time they were rapidly eliminated either through death following delayed activation or degeneration (Fig 2M). Activated follicles in *Ppp6c*^*F/F*^*;GCre+* ovaries only survived for a short time and none could develop to the preovulatory stage (Fig 2N).

The histological analysis suggested that absence of PP6c in oocytes caused defects in follicular activation and growth. To confirm these observations, we performed immunostaining of the germ cell marker MVH (mouse VASA homolog) on 2-month-old ovarian sections. As shown in S2A Fig, in normal control ovaries, primordial follicles were mostly scattered around the cortical region whereas in ovaries of adult *Ppp6c*^*F/F*^*;GCre+* mice a significant number of primordial follicles remained in clusters (S2B Fig), indicating abnormal development of primordial follicles. This finding confirmed that the natural incidence of follicular activation after puberty was disrupted by *Gdf9-Cre* mediated *Ppp6c* deletion. At 2 months-of-age, although *Ppp6c*^*F/F*^*;GCre+* mice still had a large number of growing follicles, these follicles failed to mature and ovulate. As shown by TUNEL assay on ovarian sections, increased granulosa cell apoptosis and follicle atresia (yellow arrowheads, S2D Fig) were detected in ovaries of 2-month-old *Ppp6c*^*F/F*^*;GCre+* mice compared to ovaries in control mice (S2C Fig). Furthermore, when we tried to stimulate follicle growth with exogenous PMSG, the mutant mice still could not respond normally because almost all the antral follicles initiated atresia by premature luteinization and formed numerous atretic corpora lutea (CLs) (yellow arrowheads, S2F Fig) instead of developing into preovulatory follicles (red asterisks, S2E Fig). The above data demonstrated that defective follicular development after puberty, including blocked primordial follicle activation and compromised growth of activated follicles, accounted for the infertility of *Ppp6c*^*F/F*^*;GCre+* mice.

### *Ppp6c* deletion results in retarded follicular development and oocyte death independent of AKT/mTOR but partially dependent on LKB1/AMPK pathway activity

**Fig 3:**
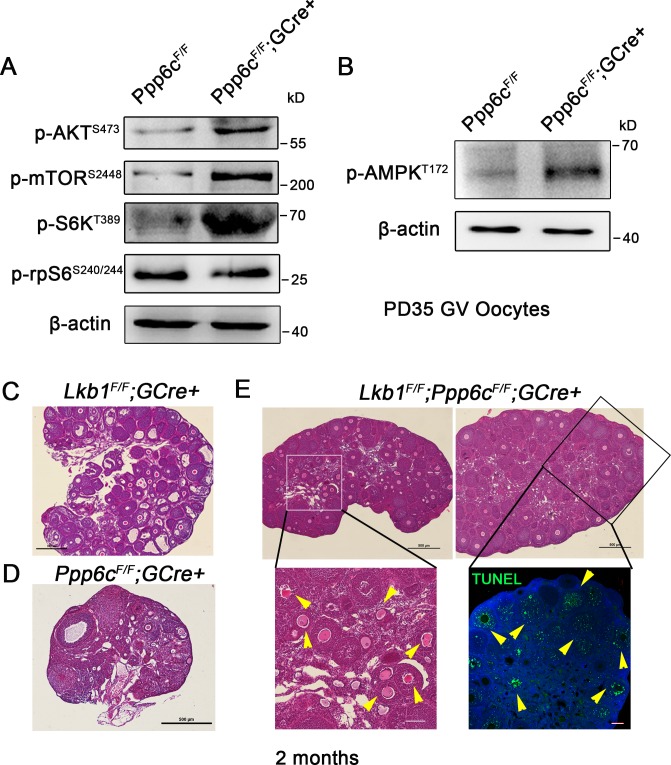
*Ppp6c* deletion results in POF independent of AKT/mTOR but partially dependent on LKB1/AMPK pathway activity. (A-B) Western blots showing up-regulated AKT/mTOR and AMPK signaling in *Ppp6c*^*F/F*^*;GCre+* oocytes. Each sample (200 GV oocytes) was collected from PD35 ovaries and immunoblotted for p-AKT, p-mTOR, p-S6K, p-rpS6, p-AMPK and β-actin. For each experiment, at least 5 mice of each genotype were used. Molecular mass is given in kilodaltons. (C-E) Histology of ovarian sections from 2-month-old *Lkb1*^*F/F*^*;GCre+*, *Ppp6c*^*F/F*^*;GCre+* and *Lkb1*^*F/F*^*;Ppp6c*^*F/F*^*;GCre+* females stained with hematoxylin and eosin. Magnified images of rectangular areas marked with a solid line are shown in H&E staining and TUNEL immunofluorescence staining. Yellow arrowheads point to atretic follicles. Green: TUNEL positive signal; Blue: DAPI. At least 3 mice of each genotype were used for analysis, and representative images are shown. Bar = 500 μm.

mTOR signaling is essential for oocyte survival and awakening from dormancy within primordial follicles [5, 29, 30]. Considering an analogous involvement of PP6 in TOR signaling in yeast and plants [17, 31], it is possible that PP6 maintains oocyte survival by regulating the mTOR pathway. Accordingly, we performed immunoblotting analysis with PD35 GV oocytes. Surprisingly, the activity of the AKT/mTORC1/S6K signaling pathway was significantly enhanced, as indicated by elevated levels of phosphorylated AKT (S473), phosphorylated mTOR (S2448), phosphorylated S6K (T389) in *Ppp6c*^*F/F*^*;GCre+* oocytes (Fig 3A and S3B Fig); phosphorylated rpS6 (S240/244) did not show obvious changes (Fig 3A and S3B Fig). This finding was not consistent with our phenotypes based on previous reports because enhanced AKT/mTOR signaling is responsible for the over-activation of primordial follicles in *Pten* and *Tsc1/2* mutant mouse models. In contrast, our *Ppp6c* mutant mice did not show any signs of premature activation of the entire primordial follicle pool, instead showing blockage/delay of follicular activation, although the activity of AKT/mTOR signaling was higher than in controls. Thus, up-regulation of the AKT/mTOR pathway could result from feedback effects to defective oocyte growth or local effects attributed to PP6 in regulating mTOR activity as suggested for other organisms [17, 31].

Recently, we reported that *Lkb1*^*fl/fl*^*; Gdf9-Cre* mice exhibit over-activation of primordial follicles starting from the onset of sexual maturity and defective follicle growth at later stages. The phenotypes of *Lkb1*^*fl/fl*^*; Gdf9-Cre* mice appear opposite to those of *Ppp6c*^*F/F*^*;GCre+* mice. Accordingly, examined the activity of AMPK, the main substrate of LKB1, in *Ppp6c*^*F/F*^*;GCre+* oocytes and observed that the level of phosphorylated AMPK (T172) was significantly increased (Fig 3B and S3B Fig); it is decreased in *Lkb1* mutant oocytes. To ascertain whether PP6 interacts with the AMPK pathway we generated double knockout mice for both *Lkb1* and *Ppp6c* (*Lkb1*^*F/F*^*;Ppp6c*^*F/F*^*;GCre+*). As expected, *Lkb1* deletion within a *Ppp6c* deletion background rescued the blockage of primordial follicle activation at 2 months-of-age (Fig 3E). Unanticipated was that double knockout ovaries resembled *Lkb1* mutant ovaries (Fig 3C) by exhibiting large sizes and over-activation of primordial follicles at 2 months-of-age. One difference, however, was that growth of activated follicles in double knockout ovaries was slower with secondary follicles containing unhealthy oocytes and showing apoptotic signals indicating extensive follicle atresia at 2 months-of-age (yellow arrowheads, Fig 3E), which is similar to 2-month-old *Ppp6c*^*F/F*^*;GCre+* ovaries (Fig 3D). In contrast, most activated follicles reached the antral follicle stage in *Lkb1* mutant ovaries at the same age (Fig 3C). These results showed that knockout of *Lkb1* could partially rescue the follicle development phenotype of the PP6c mutant ovaries, which strongly suggested involvement of AMPK in follicle development and PP6c participating in regulating the AMPK pathway. However, knockout of *Lkb1* did not rescue *Ppp6c*^*F/F*^*;GCre+* oocytes from death, suggesting that PP6 might not only regulate primordial follicle activation but also maintain survival of oocytes within primordial follicles. Therefore, we concluded that there were additional reasons for the PP6c mutant phenotype and pursued this possibility as described below.

### *Ppp6c* deletion results in increased level of γH2AX and abolishes DNA damage response in oocytes

**Fig 4:**
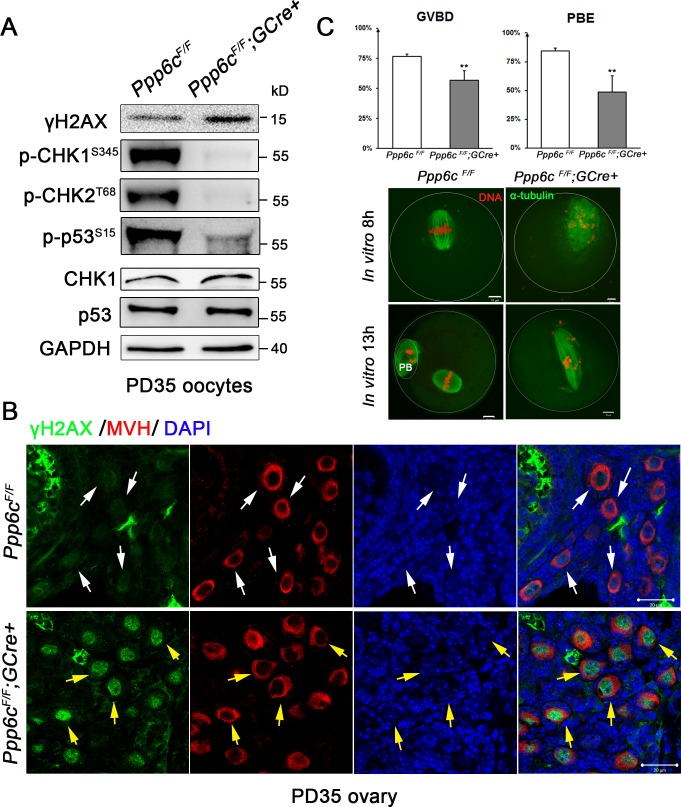
*Ppp6c* deletion results in increased level of γH2AX and abolished DNA damage response pathway in oocytes. (A) Western blots showing up-regulated level of γH2AX and down-regulated CHK1/2-p53 pathway. Level of GAPDH was used as internal controls. Molecular mass is given in kilodaltons. Oocytes were isolated from ovaries of PD35 mice and used for western blot. For each lane, 200 GV oocytes were used. For each experiment, at least 5 mice of each genotype were used. (B) Immunofluorescent staining of 2-month-old ovarian sections showing increased γH2AX in *Ppp6c*^*F/F*^*;GCre+* oocytes. Green: γH2AX; Red: MVH; Blue, DAPI. White arrows point to nucleus of control oocytes; yellow arrows point to nucleus of mutant oocytes. Bar = 20 μm. At least 3 mice of each genotype were used for analysis, and representative images are shown. (C) Decreased incidence of GVBD and PBE of *Ppp6c*^*F/F*^*;GCre+* oocytes. PD35 GV oocytes were isolated and matured *in vitro*, oocytes that resumed meiosis I (GVBD) and extruded the first polar body (PBE) were counted at 4 h and 13 h, respectively. Data are shown as mean ± SEM. *P< 0.05; **P< 0.01. Representative images of immunostaining for DNA (red) and α-tubulin (green) showing abnormal spindle assembly and aberrant chromosome alignment in *Ppp6c*^*F/F*^*;GCre+* oocytes at 8 h and 13 h, respectively. Bar = 10 μm. *In vitro* maturation experiments were repeated at least three times.

Because PP6 is involved in the DNA damage response via its ability to dephosphorylate γH2AX and antagonize DNA-dependent protein kinase (DNA-PK) [18, 19] and unrepaired meiotic or induced DNA double-strand breaks (DSBs) could cause oocyte elimination and female infertility by triggering DNA damage response pathway [32], we wondered if loss of PP6c leads to DNA damage in our case. Thus, we collected oocytes from PD35 ovaries and performed western blot analysis. As shown in Fig 4A and S3 Fig, the levels of γH2AX were significantly elevated in mutant oocytes indicating accumulated DSBs. However, the DNA damage response pathway was significantly reduced because the activity of CHK1/2-p53 signaling cascade was much lower than in controls (Fig 4A and S3 Fig). We also confirmed accumulation of γH2AX in small oocytes by immunofluorescence analysis. As indicated in Fig 4B, mutant ovaries contained more and higher nuclear signals of γH2AX within primordial follicles (yellow arrows) when compared to controls (white arrows). Oocyte maturation *in vitro* of mutant oocytes was also compromised. As shown in Fig 4C, the incidence of GVBD (56.7±7.9%) and PBE (48.7±14.2%) were lower than controls (76.6±2.0%; 84.2±2.6%, respectively). Moreover, after 8 h of *in vitro* maturation of *Ppp6c*^*F/F*^*;GCre+* oocytes spindles were disorganized with scattered chromosomes, in contrast to the well-organized MI spindles with chromosomes all aligned at the equatorial plate in *Ppp6c*^*F/F*^ oocytes. Even after 13 h of *in vitro* culture, when control oocytes had extruded the first polar body, most mutant oocytes still showed defective spindle organization and aberrant chromosome alignment, and could not complete meiosis I successfully. Taken together, these data demonstrate that loss of PP6c resulted in DSBs accumulation and severely impaired oocyte quality but deactivated the DNA damage response pathway in oocytes until puberty, which could explain why primordial follicle activation is delayed and mutant oocytes are damaged but still survive until 2 months postpartum.

### PP6c-deficient oocytes are susceptible to exogenous DNA damage

Depleting PP6c sensitizes cells to induced DNA damage [19, 33]. Thus, we speculated that this susceptibility also exists in our PP6c-deficient oocytes and leads to eventual oocyte elimination. Because endogenous DNA damage might be low and long-term, mutant oocytes would wait for repair first and then die slowly within 6 months postpartum. Experimentally increasing DNA damage in mutant oocytes could therefore trigger more rapid apoptosis and accelerate oocyte elimination if PP6c-deficient oocytes are defective in mounting a DNA damage response.

**Fig 5:**
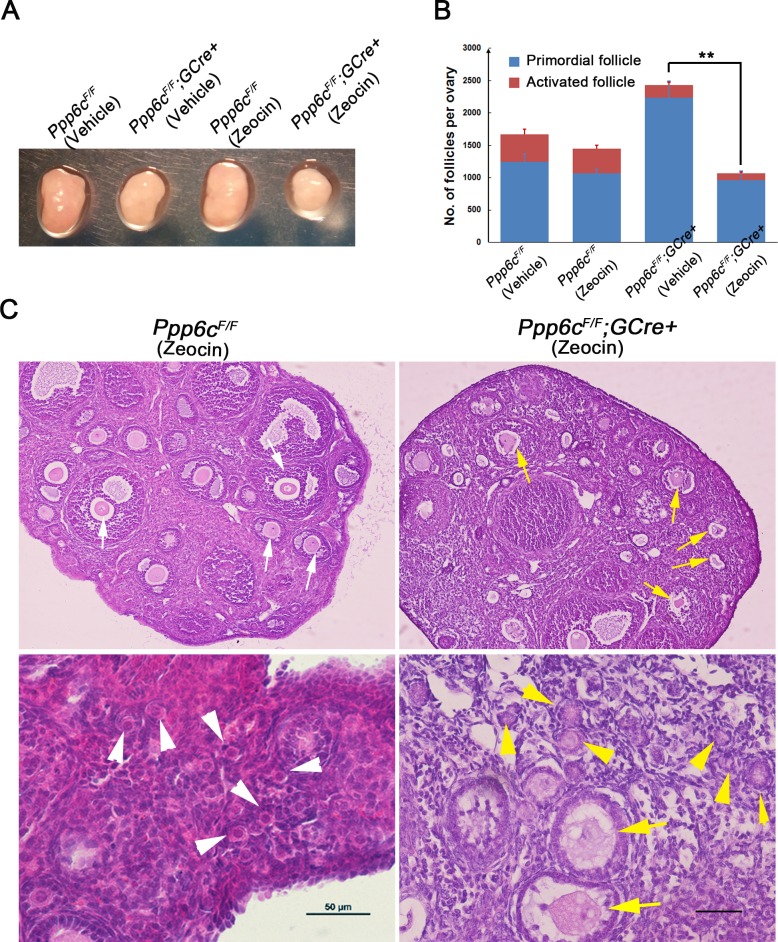
PP6c-deficient oocytes are susceptible to induced DNA damage. (A) Morphology of ovaries from *Ppp6c*^*F/F*^ and *Ppp6c*^*F/F*^*;GCre+* mice treated with zeocin or vehicle. At least 3 mice of each genotype were used for analysis, and representative images are shown. (B) Numbers of follicles including activated follicles and primordial follicles in ovaries from 2-month-old *Ppp6c*^*F/F*^ and *Ppp6c*^*F/F*^*;GCre+* mice treated with zeocin or vehicle. For each group, at least 3 mice were used for analysis. Data are shown as mean ± SEM. **P< 0.01. (C) Histology of ovaries from 2-month-old *Ppp6c*^*F/F*^ and *Ppp6c*^*F/F*^*;GCre+* mice after zeocin treatment. For each group, at least 3 mice were used for analysis. White arrows show healthy growing follicles, white arrowheads show healthy primordial follicles; yellow arrows show atretic growing follicles, yellow arrowheads show atretic primordial follicles. Bar = 50 μm.

To test this proposal, zeocin was used to induce DSBs *in vivo* by intraperitoneal injection. Forty mg of zeocin was injected per mouse once every day for 5 days [34] after which the mice were allowed 5 days of recovery and then sacrificed around 2 months postpartum. Typically, *Ppp6c*^*F/F*^*;GCre+* ovaries were smaller than the *Ppp6c*^*F/F*^ ones at 2 months-of-age; however, after zeocin treatment, mutant ovaries were even smaller whereas the size of control ovaries was similar to untreated ones (Fig 5A), suggesting that oocyte elimination was faster after zeocin treatment. This conclusion was confirmed by histological analysis of ovaries and follicle counting. After zeocin treatment, the number of primordial follicles in mutant ovaries (~57%) as well as the number of activated follicles (~44%) decreased dramatically compared to those of untreated mutant ovaries; in control groups, treated ovaries also showed fewer numbers of follicles compared to untreated ones, but these changes were not significant (Fig 5B). Consistently, as shown in Fig 5C, treated mutant ovaries contained more atretic follicles (yellow arrows), with many primordial follicles devoid of oocytes (yellow arrowheads), whereas control ovaries had plenty of healthy-looking growing follicles (white arrows) and primordial follicles (white arrowheads). The above observations showed that in response to induced DNA damage, *Ppp6c*^*F/F*^*;GCre+* ovaries showed no primordial follicle arrest but oocyte death and follicle depletion, indicating that PP6c-deficient oocytes were more sensitive to DNA damage.

**Fig 6:**
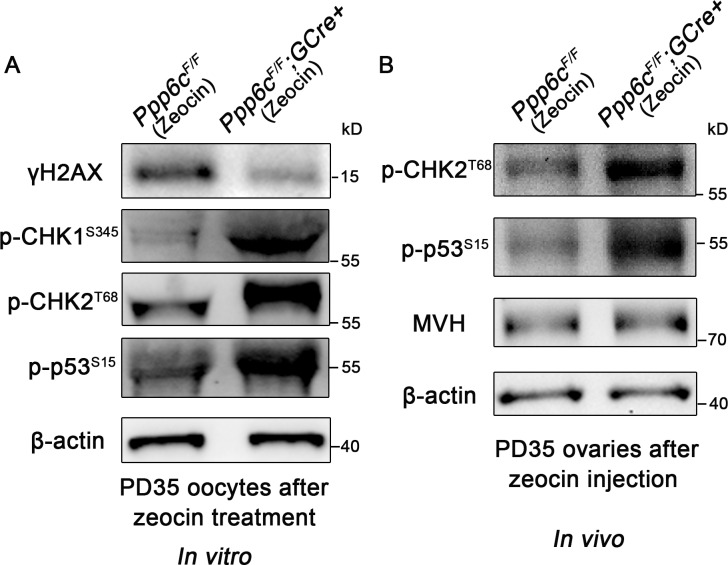
DNA damage response pathway activity is enhanced in PP6c-deficient oocytes after zeocin treatment. (A) Western blots showing up-regulated CHK1/2-p53 pathway activity in zeocin-treated *Ppp6c*^*F/F*^*;GCre+* oocytes. Level of β-actin was used as internal controls. Molecular mass is given in kilodaltons. GV oocytes were isolated from ovaries of PD35 mice and treated with zeocin *in vitro*. For each lane, 200 GV oocytes were used. For each experiment, at least 5 mice of each genotype were used. (B) Western blots showing up-regulated CHK2-p53 pathway activity in zeocin-treated *Ppp6c*^*F/F*^*;GCre+* ovaries. Level of β-actin was used as internal controls. Molecular mass is given in kilodaltons. Ovary lysates were prepared from ovaries of PD35 mice after zeocin treatment *in vivo*. For each lane, 30 μg proteins were loaded. For each experiment, at least 3 mice of each genotype were used.

To investigate the molecular causes for the results described above, we performed *in vitro* zeocin treatment in PD35 GV oocytes. GV oocytes were treated with zeocin (200 μg/ml for 1 h), then washed and cultured in M2 medium containing 2.5 μM milrinone overnight for recovery. These GV oocytes were collected for western blot analysis. As shown in Fig 6A and S4 Fig, in comparison to *Ppp6c*^*F/F*^ oocytes after treatment, *Ppp6c*^*F/F*^*;GCre+* oocytes showed lower levels of γH2AX but a highly active CHK1/2-dependent DNA damage checkpoint response, with p53-induced cell apoptosis. We also performed *in vivo* zeocin treatment in young mice (5 days of zeocin injection and 5 days of recovery) and collected ovaries for western blot at ~PD35 when mutant ovaries still had similar numbers of follicles as controls (Fig 6B and S4 Fig). The levels of MVH, a marker of germ cells, were similar in both groups indicating mutant ovaries still contained comparable numbers of oocytes to controls. Mutant ovaries, however, showed an enhanced CHK2-p53 DNA damage response pathway activity, suggesting PP6c-deficient oocytes could not repair induced DNA damage and would die eventually. Based on the above results, the main cause for the PP6c depletion phenotype appeared to be an increased susceptibility to DNA damage of PP6c-deficient oocytes.

Collectively, these findings support the notion that PP6 is a critical regulator for oocyte survival and follicle development by restraining phosphorylation of H2AX to normal levels and participating in AMPK pathway regulation.

## Discussion

In female reproduction, production of high quality eggs requires both successful follicular development and precise completion of oocyte meiosis. Previously, we studied the roles of PP6c in meiosis completion by crossing *Ppp6c*^*F/F*^ mice with *Zp3-Cre* mice. By crossing *Ppp6c*^*F/F*^ mice with *Gdf9-Cre* mice to generate mutant mice with a specific deletion of *Ppp6c* in oocytes from the primordial follicle stage we were able to investigate the roles of PP6c in follicular development. We find that *Ppp6c* mutant female mice show defective folliculogenesis and are infertile. Importantly, PP6c depletion caused persistent phosphorylation of H2AX. Thus, susceptibility to DNA damage and defective DNA repair mechanisms turned out to be the main underlying causes for the observed infertility. In addition, PP6c may control follicular activation by regulating the AMPK pathway.

During embryonic development, primordial germ cells in female mammals enter meiosis I and finish a crucial process called synapsis that requires homologous recombination (HR), a high-fidelity DNA double-strand break (DSB) repair process. Aberrant homolog synapsis or DSB repair triggers checkpoints that eliminate defective meiotic oocytes [35–37]. Loss of oocytes defective in DSB repair occurs soon after birth, which is controlled by the DNA damage checkpoint including the CHK2-p53/p63 pathway [32]. Oocytes are subsequently arrested at the dictyate stage of prophase I in the form of dormant oocytes enclosed in primordial follicles [38]. Such prophase I arrest usually takes weeks or months, or even longer in mice, and after primordial follicular activation, undergo a prolonged period of follicular growth before meiosis resumption and ovulation [39, 40]. The lengthy dormancy and growth of oocytes makes maintenance of genomic integrity during follicular development more challenging and important for generating healthy gametes. However, the underlying molecular mechanisms to protect genomic DNA after embryonic HR and DSB repair remained undiscovered. The DNA damage checkpoint usually acts around the time oocytes enter meiotic arrest but presumably persists, because resting primordial follicles are highly sensitive to ionizing radiation (IR) [41]. In our study, oocyte-specific knockout of PP6c from primordial follicle stages results in increased γH2AX in arrested oocytes and the whole germ cell pool is then progressively eliminated by DNA damage checkpoint pathway within 6 months postpartum. These findings make PP6 a competitive candidate for safeguarding genomic DNA integrity of female germ cells during the long prophase I arrest.

As noted above, PP6 is implicated in the cell response to DNA damage. The phosphorylated form of H2AX on S139 (γH2AX) is a marker of DSBs. PP6c exhibits phosphatase activity against γH2AX in *in vitro* phosphatase assays. In human cancer lines, depletion of PP6c or PP6R2 leads to persistent high levels of γH2AX after DNA damage and defective homology-directed repair (HDR) [19]. PP6c is recruited to DSB sites by DNA-PK, and PP6 is also required for efficient activation of DNA-PK, which is essential for non-homologous end joining (NHEJ)-mediated repair of DSBs [18, 42]. A recent study also showed that *Ppp6c*-deficient mouse keratinocytes exhibit a high frequency of both p53- and γH2AX-positive cells, suggestive of DNA damage, as well as up-regulated expression of p53, PUMA, BAX, and cleaved caspase-3 proteins following UVB irradiation [33]. Our *in vivo* data show that absence of PP6c also leads to higher levels of γH2AX (Fig 4) and defective DNA repair in oocytes, especially massive oocyte death after induced DNA damage (Figs 5 and 6), suggesting that PP6 has a conserved role in DNA damage response, which is essential for gamete production and fertility maintenance.

As members of the well-known PP2A-like subfamily, PP6 shares common features with PP2A or PP4. As phosphatases, they all are involved in a diverse set of biological pathways due to their wide range of substrates. Until now, PP6 was implicated in regulation of DNA damage response, cell cycle progression, apoptosis, pre-mRNA splicing, signaling through the mTOR pathway and Hippo pathway, and others [19, 22, 23, 25, 28, 33]. Among these multiple functions, mTOR pathway regulation was first considered as the potential cause of the phenotype in our study. mTOR signaling regulates follicular activation and oocyte survival because oocyte-specific deletion of its upstream genes, *Pten* or *Tsc1/2*, lead to premature activation of the entire primordial follicle pool, resulting in POF due to enhanced mTORC1-S6K-rpS6 signaling [5, 29, 30]. Although PP6c-deficient oocytes also show similar enhanced AKT/mTOR signaling, the ovarian phenotype of *Ppp6c* mutant mice is not similar at all, because PP6c mutant ovaries show blocked/delayed follicular activation instead of premature activation, also at later time points.

Although the AKT/mTOR pathway is activated in PP6c mutant ovaries, primordial follicles are not activated, perhaps because the downstream effectors of mTOR pathway are not responding. As seen from the western blot results (Fig 3A), the activities of the AKT/mTORC1/S6K signaling are significantly enhanced in *Ppp6c*^*F/F*^*;GCre+* oocytes, but as the downstream effector that enhances protein translation, rpS6 does not show an obvious change of activity. Thus, the effects of AKT/mTOR pathway activation are somehow blocked at the execution phase and therefore do not activate primordial follicles in mutant ovaries. In light of these findings, we turned to another important folliculogenesis regulator, the LKB1-AMPK pathway.

Our previous study reported that *Lkb1* mutant female mice show over-activation of primordial follicles after puberty [6], at a similar time point as that of *Ppp6c* mutant mice. Because Western blot results also show up-regulated p-AMPK in PP6c-deficient oocytes, opposite to that in LKB1-deficient oocytes, we generated double knockout of *Lkb1* and *Ppp6c* in oocytes to try to rescue the phenotypes of *Ppp6c* mutant mice. Indeed, the blocked/delayed follicular activation was rescued, which means mis-regulation of AMPK pathway could be a partial reason for the PP6c mutant phenotypes. Moreover, the double knockout ovaries show over-activation of primordial follicles, more similar to *Lkb1* single knockout, but accelerated oocyte death and slower follicle growth, suggesting that absence of PP6c might affect oocyte quality and survival more directly than just control follicular activation. Thus, PP6’s role in DNA damage response could be the main cause. Consistent with this proposal is that PP6c depletion caused increased γH2AX, a marker of DSBs, and defective DNA repair in oocytes, with accelerated oocyte death with induced DNA damage. Interestingly, oocyte defects resulting from PP6c depletion are relatively low in natural circumstances, and oocyte death occurs only when both endogenous and exogenous harm accumulated with time to a certain degree, which could explain why the whole oocyte elimination process took up to 6 months in *Ppp6c* mutant ovaries. Thus, PP6c could control oocyte quality through its role in DDR pathway as well as regulate follicular activation through participating in the AMPK pathway. Nevertheless, we cannot exclude other possibilities, e.g., regulation of pre-mRNA splicing and Hippo pathway, that could also contribute to the phenotypes in our mutant mouse model.

Female meiosis is error-prone in humans. Our previous study reported that *Zp3-Cre* mediated PP6c depletion in growing oocytes leads to defective MII spindle function and unfaithful chromatid segregation in meiosis II without affecting folliculogenesis, indicating that PP6 can act as antagonizer to oocyte aneuploidy during the MII exit. Here we demonstrate that *Gdf9-Cre* mediated PP6c depletion in dormant oocytes causes defective folliculogenesis and massive germ cell elimination at early stages, indicating that PP6 can also safeguard oocyte genomic integrity and regulate folliculogenesis during the long prophase I arrest. Furthermore, isolated GV oocytes from *Ppp6c*^*F/F*^*;GCre+* mice before POF occurs show severely impaired *in vitro* maturation because of DNA damage, in sharp contrast to the unaffected meiotic maturation progress of *Ppp6c*^*F/F*^*;ZCre+* oocytes. Although these two knockout mouse models are both oocyte-specific knockouts, they exhibit completely different phenotypes that presumably reflect differences between timing of *Zp3-Cre* and *Gdf9-Cre* expression. Both ZP3 and GDF9 are specifically expressed in oocytes. The synthesis of ZP3 starts in primary follicles from PD5, reaches a maximum in growing follicles, and decreases in full-grown oocytes, which makes *Zp3-Cre* only suitable for deletion of gene expression in oocytes from primary follicle stages on. However, *Gdf9-Cre* is expressed in oocytes from primordial follicle stage. This difference in expression is presumably why *Ppp6c*^*F/F*^*;GCre+* mice display primordial follicle defects whereas *Ppp6c*^*F/F*^*;ZCre+* mice do not.

In summary, we provide evidence that PP6 acts as a critical guard of genomic integrity in lengthy prophase I arrest of oocytes and is an indispensable regulator of folliculogenesis, and thus female fertility. Our data may provide valuable information for the design of therapeutics for POF.

## Materials and Methods

### Ethics statement

Animal care and handling were conducted according to the guidelines of the Animal Research Committee of the Institute of Zoology, Chinese Academy of Sciences. The institutional committee which is licensed by Beijing Municipal Experimental Animal Administration approved this study.

### Mice

Mice lacking *Ppp6c* in oocytes (referred to as *Ppp6c*^*F/F*^*;GCre+*) were generated by crossing *Ppp6c*^*F/F*^ mice [28] with *Gdf9-Cre* mice. Both transgenic mouse lines have C57BL/6J genomic background. The mice were housed under controlled environmental conditions with free access to water and food. Light was provided between 08:00 and 20:00.

### Reagents and antibodies

Commercial antibodies were used to detect PPP6C (rabbit, A300-844A; Bethyl Laboratories, Inc.), α-tubulin (mouse, DM1A; Sigma-Aldrich), MVH (rabbit, ab13840; Abcam), γH2AX (rabbit, 9718; Cell Signaling Technology, Inc.), p-CHK1 (S345) (rabbit, BS4041; Bioworld Technology, Inc.), p-CHK2 (T68) (rabbit, BS4043; Bioworld Technology, Inc.), p-p53 (S15) (rabbit, 12571; Cell Signaling Technology, Inc.), CHK1 (rabbit, BS1052; Bioworld Technology, Inc.), p-AKT (S473) (rabbit, 4060; Cell Signaling Technology, Inc.), p-AMPK (T172) (rabbit, 2535; Cell Signaling Technology, Inc.), p-mTOR (S2448) (rabbit, 5536; Cell Signaling Technology, Inc.), p-S6K (T389) (rabbit, 9234; Cell Signaling Technology, Inc.), p-rpS6 (S240/244) (Rabbit, 5364; Cell Signaling Technology, Inc.), GAPDH (rabbit, 5174; Cell Signaling Technology, Inc.) and β-actin (mouse, sc-47778, Santa Cruz). Secondary antibodies were purchased from ZhongShan Golden Bridge Biotechnology Co., LTD (Beijing).

### Histological analysis, immunostaining and TUNEL assay

Ovaries used for histological analysis were collected from adult female mice. They were fixed in 4% paraformaldehyde (pH 7.5) overnight at 4°C, dehydrated, and embedded in paraffin. Paraffin-embedded ovaries were sectioned at a thickness of 8-μm for hematoxylin and eosin (H&E) staining. One or both ovaries from more than three mice of each genotype were used for the analysis. Paraffin-embedded ovarian tissue sections were deparaffinized, immersed in retrieval solution (10 mM sodium citrate), heated in an autoclave, blocked with 10% normal goat serum, and then incubated overnight with primary antibodies (anti-MVH and anti-γH2AX at 1:200 dilution). For immunofluorescence, localization of the primary antibody was performed by incubation of the sections with the corresponding secondary antibodies (Invitrogen) at 1:500 dilution for 1h at room temperature. Finally, nuclei were stained with DAPI. For immunohistochemistry, the Vecta stain ABC kit (Vector Laboratories, CA, USA) was used to detect the signal of primary antibody. Analysis of apoptosis in ovarian follicles was carried out by TUNEL assay using the ApopTag Plus in situ apoptosis detection kit (Chemicon International, Temecula, CA, USA). At least three different samples from each genotype were analyzed in parallel.

Oocytes for immunofluorescent staining were fixed in 4% paraformaldehyde in PBS for 30 min at room temperature. The fixed oocytes were then transferred to membrane permeabilization solution (0.5% Triton X-100) for 20 min and blocking buffer (1% BSA-supplemented PBS) for 1 h. The oocytes were then incubated overnight at 4°C with FITC conjugated anti-α-tubulin at 1:2000 dilution. Nuclei were stained with DAPI. Finally, oocytes were mounted on glass slides and examined with a laser scanning confocal microscope (Zeiss LSM 780 META, Germany).

### Quantification of ovarian follicles

Quantification of ovarian follicles was performed as previously described [43]. Briefly, to count the numbers of follicles, paraffin-embedded ovaries were serially sectioned at 8-μm thickness and every fifth section was mounted on slides. Then these sections were stained with hematoxylin and eosin for morphological analysis. Ovarian follicles at different developmental stages, including primordial, primary (type 3 and type 4), secondary (type 5) and antral follicles (type 6 and type 7) were counted in collected sections of an ovary, based on the well-accepted standards established by Peterson and Peters [44]. In each section, only those follicles in which the nucleus of the oocyte was clearly visible were scored and the cumulative follicle counts were multiplied by a correction factor of 5 to represent the estimated number of total follicles in an ovary.

### Natural ovulation

For the natural ovulation assay, 2–4 month-old female mice were mated with fertile males overnight. Successful mating was confirmed by the presence of vaginal plugs. Fertilized eggs were harvested from oviducts, counted and analyzed after removal of the cumulus mass with 1mg/ml hyaluronidase (Sigma-Aldrich) in M2 medium (Sigma-Aldrich).

### Oocyte collection and culture

GV stage oocytes were isolated from ovaries of ~PD35 female mice and cultured in M2 medium under paraffin oil at 37°C, 5% CO_2_ in air.

For *in vitro* treatment of zeocin, fully-grown GV oocytes were first treated with zeocin (200 μg/ml, Invitrogen) for 1 h in M2 medium supplemented with 2.5 μM milrinone and then blocked by the same concentration of milrinone for recovery. Oocytes were collected after 12 hours of recovery for western blot.

### Western blot analysis

Ovary lysate was prepared from minced ovaries after removal of suspended granulosa cells by centrifugation for western blot analysis. Thirty μg ovary protein or 200 oocytes were mixed with SDS sample buffer and boiled for 5 min at 100°C for SDS-PAGE. Western blot was performed as described previously [45], using antibody dilutions as below, antibodies against PPP6C, MVH, γH2AX, p-CHK1 (S345), p-CHK2 (T68), CHK1 at 1:500, antibody against p53, p-p53 (S15), p-AKT (S473), p-AMPK (T172), p-mTOR (S2448), p-S6K (T389), p-rpS6 (S240/244) at 1:1000, and antibodies against GAPDH and β-actin at 1:2000.

### *In vivo* treatment of mice with zeocin

To induce DNA DSBs *in vivo*, zeocin was injected into the abdominal cavity of female mice once every day for 5 days, and physiological saline (vehicle) was injected as control. Zeocin (100 mg/ml, Invitrogen) was diluted in physiological saline to give a final concentration of 400 mg/ml, and 0.1 ml (40 μg zeocin) was injected per mouse. At least 3 mice were injected in each group. Mice were sacrificed 5 days after injection and ovaries were fixed for histological analysis or lysed for western blot.

### Breeding assay

In breeding assays, *Ppp6c*^*F/F*^ and *Ppp6c*^*F/F*^*;GCre+* female mice with sexual maturity were continually mated to *Ppp6c*^*F/F*^ male mice with known fertility for 6 months. Cages were checked daily for counting the number of litters and pups.

### Statistical analysis

All experiments were repeated at least three times. Student’s t test was used for statistical analysis and performed using SPSS. Data were expressed as mean ± SEM and values are statistically significant at *P<0.05; **P<0.01.

## Supporting Information

S1 Fig (A) Histology of ovarian sections from 1-month-old *Ppp6c*^*F/F*^ and *Ppp6c*^*F/F*^*;GCre+* females stained with hematoxylin and eosin. At least 3 mice of each genotype were used for analysis, and representative images are shown. Bar = 500 μm. (B-F) Shown are the quantifications of numbers of different types of follicles per ovary at the age of 2 months, 3 months, 4 months and 6 months, respectively. Primordial (Pri), type 3 (T3), type 4 (T4), type 5 (T5), type 6 (T6) and type 7 (T7) follicles were counted. The numbers of analysed mice are indicated (n). Data are shown as mean ± SEM. *P< 0.05; **P< 0.01. (G) Numbers of total follicles in ovaries of 1-month (1 mo), 2-month (2 mo), 3-month (3 mo), 4-month (4 mo) and 6-month (6 mo)-old *Ppp6c*^*F/F*^ and *Ppp6c*^*F/F*^*;GCre+* females. Data are shown as mean ± SEM.
(TIF)

S2 Fig Loss of PP6c causes primordial follicle arrest and growing follicle atresia. (A-B) Immunofluorescent staining of ovarian sections for germ cell marker (MVH) showing primordial follicle clusters in 2-month-old *Ppp6c*^*F/F*^*;GCre+* female mice. Red, MVH; Blue, DNA. Bar = 100 μm. At least 3 mice of each genotype were used for analysis, and representative images are shown. (C-F) TUNEL assays showing follicle atresia in 2-month-old ovaries (C-D) and after injection of PMSG (E-F) of the indicated genotypes. Red asterisks indicate preovulatory follicles. Yellow arrowheads point to atretic follicles. Bar = 500 μm. At least 3 mice of each genotype were used for analysis, and representative images are shown.
(TIF)

S3 Fig (A) Western blots scanned in full length showing PP6c depletion, up-regulated AKT/mTOR signaling, upregulated AMPK pathway, increased level of γH2AX and downregulated CHK2-p53 pathway in *Ppp6c*^*F/F*^*;GCre+* oocytes. Molecular mass is given in kilodaltons. (B) Relative intensity of PP6c, p-AKT (S473), p-mTOR (S2448), p-S6K (T389), p-rpS6 (S240/244), p-AMPK (T172), γH2AX, p-CHK1 (S345), p-CHK2 (T68), p-p53 (S15), CHK1 and p53 with PD35 GV oocytes from *Ppp6c*^*F/F*^ and *Ppp6c*^*F/F*^*;GCre+* mice. Data are shown as mean ± SEM. *P<0.05, **P< 0.01.
(TIF)

S4 Fig (A-B) Western blots scanned in full length showing upregulated CHK2-p53 pathway activity in zeocin-treated PD35 *Ppp6c*^*F/F*^*;GCre+* oocytes and PD35 ovaries. Molecular mass is given in kilodaltons. (C) Relative intensity of γH2AX, p-CHK1 (S345), p-CHK2 (T68), p-p53 (S15) and MVH with PD35 GV oocytes and PD35 ovary protein extract from *Ppp6c*^*F/F*^ and *Ppp6c*^*F/F*^*;GCre+* mice after zeocin treatment. Data are shown as mean ± SEM. *P<0.05, **P< 0.01.
(TIF)
