# Supplementary figures and images for: Protein Phosphatase 6 Protects Prophase I-Arrested Oocytes by Safeguarding Genomic Integrity
Source: PLoS Genet. 2016 Dec 8;12(12):e1006513. doi: 10.1371/journal.pgen.1006513 (PMC5179128; doi:10.1371/journal.pgen.1006513)

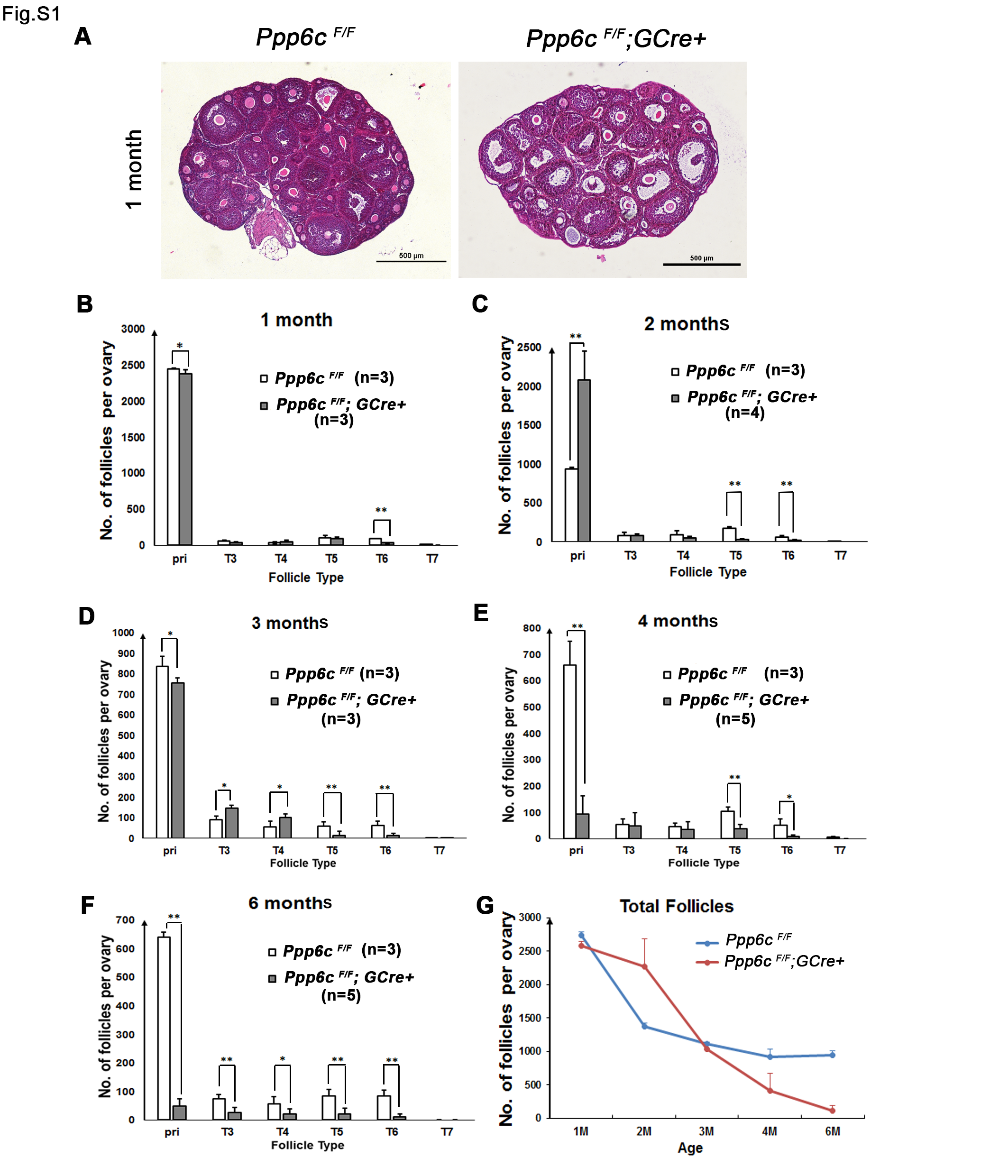

Supplement: S1 Fig — (A) Histology of ovarian sections from 1-month-old Ppp6cF/F and Ppp6cF/F;GCre+ females stained with hematoxylin and eosin. At least 3 mice of each genotype were used for analysis, and representative images are shown. Bar = 500 μm. (B-F) Shown are the quantifications of numbers of different types of follicles per ovary at the age of 2 months, 3 months, 4 months and 6 months, respectively. Primordial (Pri), type 3 (T3), type 4 (T4), type 5 (T5), type 6 (T6) and type 7 (T7) follicles were counted. The numbers of analysed mice are indicated (n). Data are shown as mean ± SEM. *P< 0.05; **P< 0.01. (G) Numbers of total follicles in ovaries of 1-month (1 mo), 2-month (2 mo), 3-month (3 mo), 4-month (4 mo) and 6-month (6 mo)-old Ppp6cF/F and Ppp6cF/F;GCre+ females. Data are shown as mean ± SEM. (TIF) [file pgen.1006513.s001.tif]

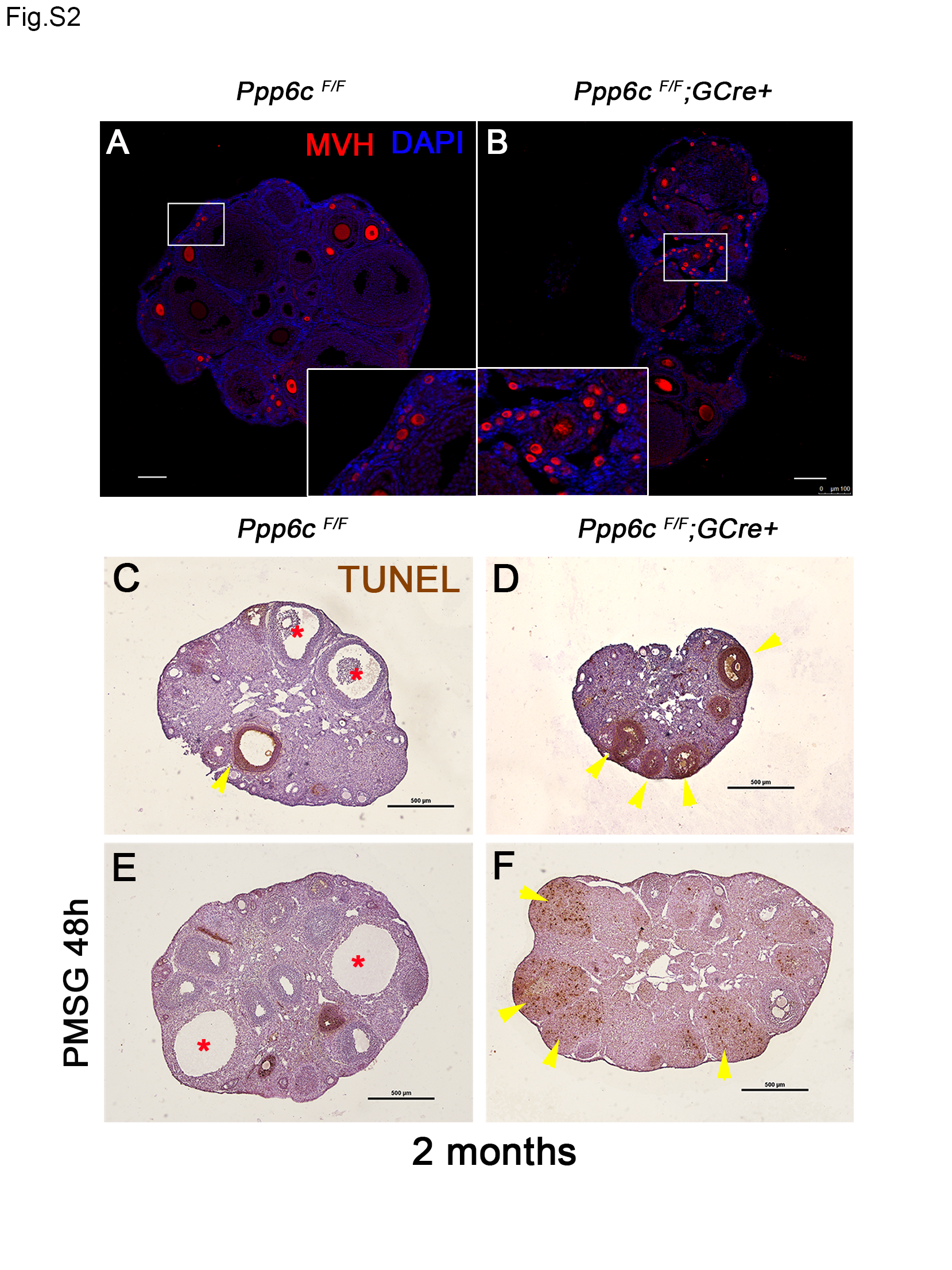

Supplement: S2 Fig — (A-B) Immunofluorescent staining of ovarian sections for germ cell marker (MVH) showing primordial follicle clusters in 2-month-old Ppp6cF/F;GCre+ female mice. Red, MVH; Blue, DNA. Bar = 100 μm. At least 3 mice of each genotype were used for analysis, and representative images are shown. (C-F) TUNEL assays showing follicle atresia in 2-month-old ovaries (C-D) and after injection of PMSG (E-F) of the indicated genotypes. Red asterisks indicate preovulatory follicles. Yellow arrowheads point to atretic follicles. Bar = 500 μm. At least 3 mice of each genotype were used for analysis, and representative images are shown. (TIF) [file pgen.1006513.s002.tif]

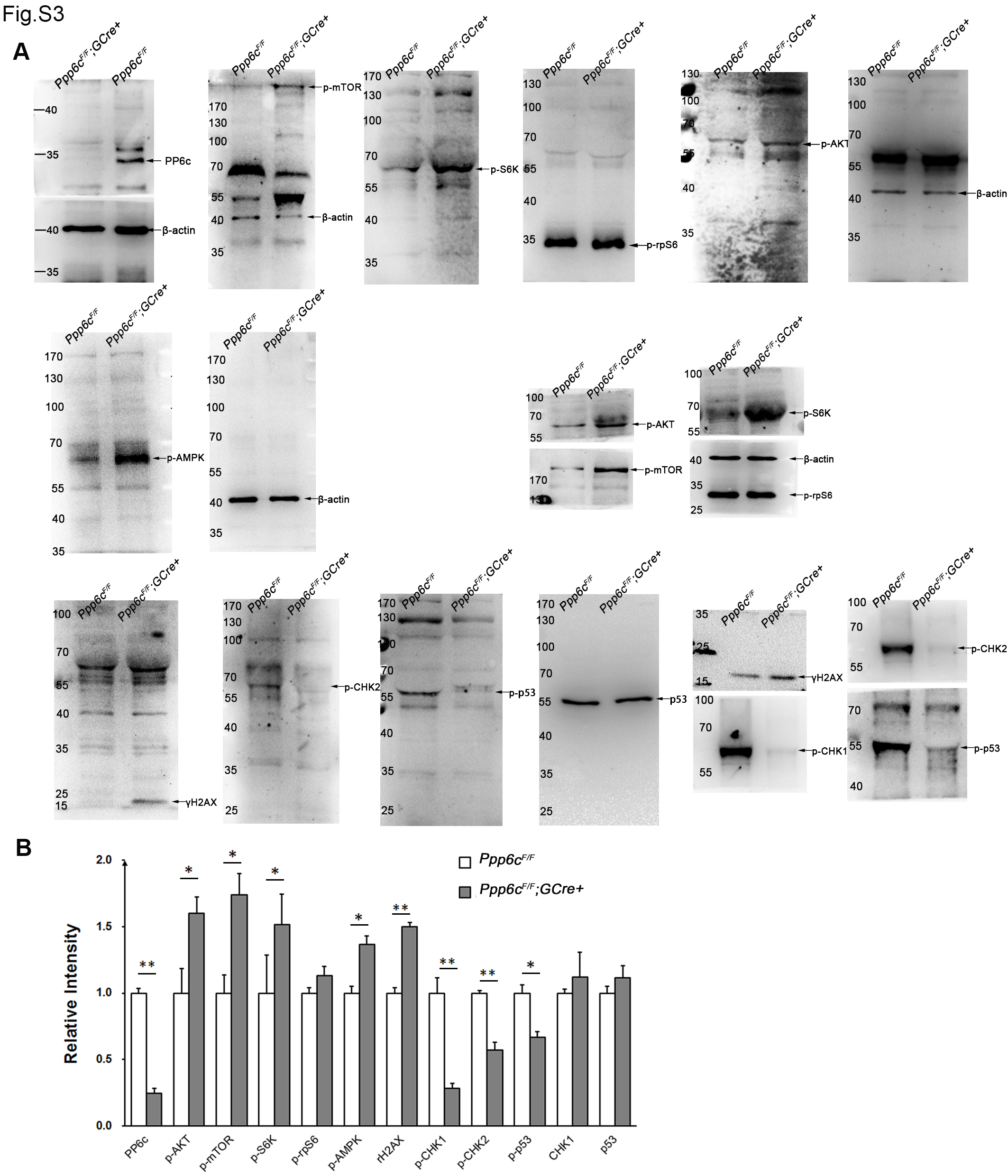

Supplement: S3 Fig — (A) Western blots scanned in full length showing PP6c depletion, up-regulated AKT/mTOR signaling, upregulated AMPK pathway, increased level of γH2AX and downregulated CHK2-p53 pathway in Ppp6cF/F;GCre+ oocytes. Molecular mass is given in kilodaltons. (B) Relative intensity of PP6c, p-AKT (S473), p-mTOR (S2448), p-S6K (T389), p-rpS6 (S240/244), p-AMPK (T172), γH2AX, p-CHK1 (S345), p-CHK2 (T68), p-p53 (S15), CHK1 and p53 with PD35 GV oocytes from Ppp6cF/F and Ppp6cF/F;GCre+ mice. Data are shown as mean ± SEM. *P<0.05, **P< 0.01. (TIF) [file pgen.1006513.s003.tif]

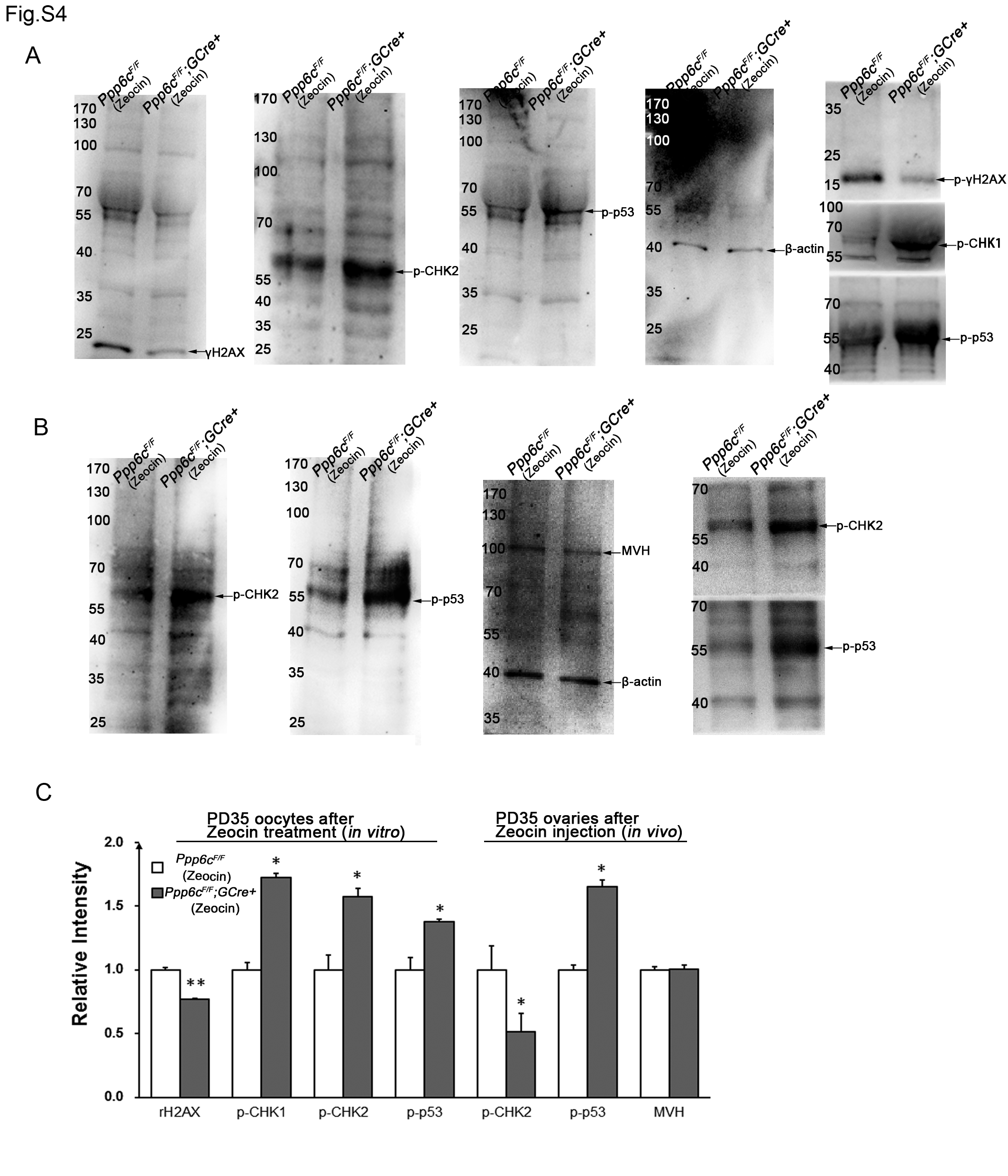

Supplement: S4 Fig — (A-B) Western blots scanned in full length showing upregulated CHK2-p53 pathway activity in zeocin-treated PD35 Ppp6cF/F;GCre+ oocytes and PD35 ovaries. Molecular mass is given in kilodaltons. (C) Relative intensity of γH2AX, p-CHK1 (S345), p-CHK2 (T68), p-p53 (S15) and MVH with PD35 GV oocytes and PD35 ovary protein extract from Ppp6cF/F and Ppp6cF/F;GCre+ mice after zeocin treatment. Data are shown as mean ± SEM. *P<0.05, **P< 0.01. (TIF) [file pgen.1006513.s004.tif]
